# Faithful animal modelling of human glioma by using primary initiating cells and its implications for radiosensitization therapy

**DOI:** 10.1038/s41598-018-32578-w

**Published:** 2018-09-21

**Authors:** Guido Frosina, Jean Louis Ravetti, Renzo Corvò, Mauro Fella, Maria Luisa Garrè, Fabrizio Levrero, Diana Marcello, Daniela Marubbi, Giovanni Morana, Michele Mussap, Carlo Emanuele Neumaier, Aldo Profumo, Alessandro Raso, Francesca Rosa, Stefano Vagge, Donatella Vecchio, Antonio Verrico, Gianluigi Zona, Antonio Daga

**Affiliations:** 1 Mutagenesis & Cancer Prevention, IRCCS Ospedale Policlinico San Martino, 16132 Genova, Italy; 2 Pathological Anatomy and Histology, IRCCS Ospedale Policlinico San Martino, 16132 Genova, Italy; 3 Radiation Oncology, IRCCS Ospedale Policlinico San Martino, 16132 Genova, Italy; 4 Department of Health Sciences (DISSAL) University of Genoa, 16132 Genova, Italy; 5 Laboratory Medicine, IRCCS Ospedale Policlinico San Martino, 16132 Genova, Italy; 6 IRCCS Istituto Giannina Gaslini, 16147 Genova, Italy; 7 Medical Physics, IRCCS Ospedale Policlinico San Martino, 16132 Genova, Italy; 8 Regenerative Medicine, IRCCS Ospedale Policlinico San Martino, 16132 Genova, Italy; 9 Department of Experimental Medicine (DIMES), University of Genova, 16132 Genova, Italy; 10 Diagnostic Imaging and Interventional Oncology, IRCCS Ospedale Policlinico San Martino, 16132 Genova, Italy; 11 Biopolymers and Proteomics, IRCCS Ospedale Policlinico San Martino, 16132 Genova, Italy; 12 Neurosurgery and Neurotraumatology, IRCCS Ospedale Policlinico San Martino, 16132 Genova, Italy; 13 Department of Neurology, Ophthalmology, Genetics and Paediatrics (DINOGMI), University of Genova, 16132 Genova, Italy

## Abstract

It has been reported that the ATM kinase inhibitor KU60019 preferentially radiosensitizes orthotopic high grade gliomas (HGG) driven by established U87 and U1242 cell lines bearing specific *TP53* mutations. We wished to determine whether those results could be extended to tumors driven by primary glioma initiating cells (GIC) that closely mimic clinical tumors. Orthotopic HGG were developed in immunodeficient non-obese diabetic-severe combined immunodeficient (NOD-SCID) mice by intracranial injection of primary GIC isolated from the adult glioblastoma COMI (acronym of patient’s name) and the pediatric anaplastic astrocytoma 239/12. Similar to the clinical tumors of origin, the orthotopic tumors COMI and 239/12 displayed different growth properties with a voluminous expansive lesion that exerted considerable mass effect on the adjacent structures and an infiltrating, gliomatosis-like growth pattern with limited compressive attitude, respectively. Significant elongations of median animal survival bearing the adult COMI tumor was observed after one KU60019 convection enhanced delivery followed by total 7.5 Gy of ionizing radiation delivered in fifteen 0.5 Gy fractions, as compared to animals treated with vehicle + ionizing radiation (105 vs 89 days; ratio: 0.847; 95% CI of ratio 0.4969 to 1.198; P:0.0417). Similarly, a trend to increased median survival was observed with the radiosensitized pediatric tumor 239/12 (186 vs 167 days; ratio: 0.8978; 95% CI of ratio: 0.5352 to 1.260; P: 0.0891). Our results indicate that radiosensitization by KU60019 is effective towards different orthotopic gliomas that faithfully mimic the clinical tumors and that multiple GIC-based animal models may be essential to develop novel therapeutic protocols for HGG transferable to the clinics.

## Introduction

High grade gliomas (HGG-WHO grades III and IV) are the most frequent gliomas accounting for almost 50% of cases in Europe^1,2^. Despite advances in surgery and radiotherapy (RT), the prognosis for HGG remains poor, with median patient’s survival of 12–14 months for grade IV [glioblastoma(GB)] and 24–60 months for grade III [anaplastic astrocytoma (AA)]^3,4^. The ineffectiveness of currently available chemotherapeutic agents and RT depends on intrinsic tumor properties including elevated infiltration capacity and resistance to therapies^5^. In particular, resistance of HGG to RT may in part be linked to temporary quiescence of subpopulations of cells driving tumor development and progression [glioma initiating cells (GIC)], in turn caused by constitutive activation of the response to DNA damage (DDR)^6,7^. Ataxia Telangiectasia Mutated (ATM) kinase is a major sensor of DNA damage and activator of DDR^8^. *In vitro* studies in our and other laboratories have shown that inhibition of ATM protein by the specific inhibitor KU60019 can flush GIC out of their quiescence and sensitize them to ionizing radiation (IR)^9–17^. *In vivo*, inhibition of ATM kinase by KU60019 has been reported as an effective radiosensitization strategy of orthotopic gliomas driven by the established U87 and U1242 cell lines bearing specific *TP53* mutations^17^. We show here that primary GIC-driven orthotopic tumors faithfully mimic the growth properties of the clinical tumors and that the *in vivo* radiosensitization and survival advantages achievable by the ATM inhibitor KU60019 can be partially extended to these models.

## Materials and Methods

### Approvals

All procedures performed in studies involving human participants were in accordance with the ethical standards of the institutional and/or national research committees and with the 1964 Helsinki declaration and its later amendments or comparable ethical standards. In particular, the adult COMI patient’s informed consent for study participation was obtained and the study was approved by the Regional Committee for Ethical Experimentation, Ospedale Policlinico San Martino, Genova, Italy (record n. P.R.216REG2015). Similarly, the 239/12 pediatric patient’s informed consent for study participation was obtained by parents and the study was approved by the Regional Committee for Ethical Experimentation with protocol n. HERBY B025041/ITCC019/HIGH GRADE GLIOMA – 001 Version Ita 01231358v01 of 25/10/2011).

All applicable international, national, and/or institutional guidelines for the care and use of animals were followed. In particular, all animal experiments were performed in compliance with guidelines approved by the Italian Ministry of Health and the committee for animal well-being in cancer research (OPBA) at Ospedale Policlinico S.Martino - Genova, Italy (Italian Ministry of Health authorization n. 1309/2015-PR). The ARRIVE (Animal Research: Reporting of *In Vivo* Experiments) guidelines were followed throughout this report^1^.

### Chemicals

The ATM inhibitor 2-[(2R, 6S)-2, 6-dimethylmorpholin-4-yl]-N-[5-(6-morpholin-4-yl-4-oxo-4H-pyran-2-yl)-9H-thioxanthen-2-yl]-acetamide (KU60019) was purchased from Selleck Chemicals (Houston, TX, USA, product code: S1570).

### Experimental design

**Figure 1:**
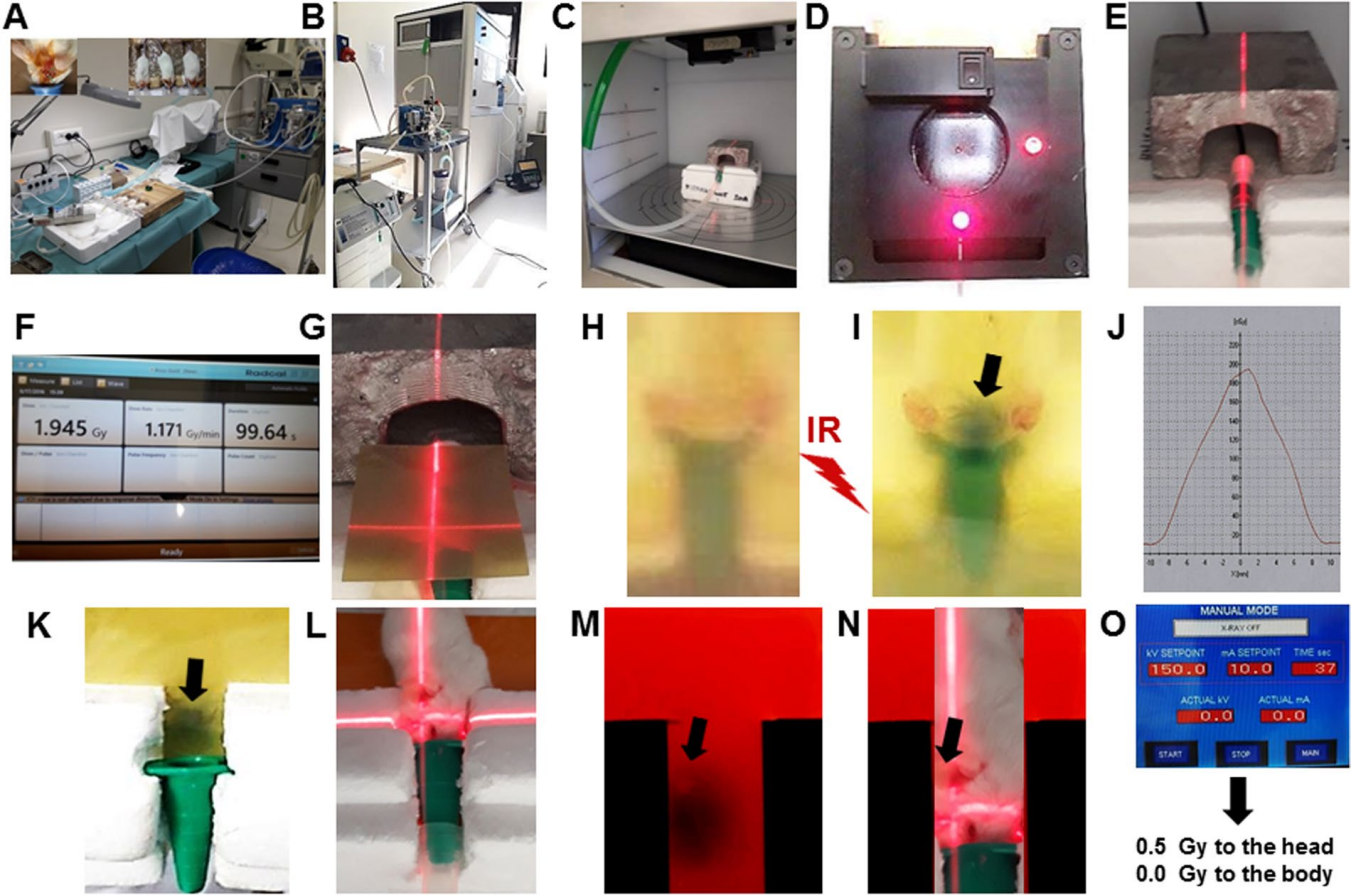
Experimental set up for multiple radiosensitizations *in vivo*. (**A**) Device i. In order to reduce toxicity during multiple radiosensitization cycles, CED was carried out under inhalation anesthesia with isoflurane as described under Materials and methods and^13^. Top left and top central insets are detail snapshots showing the location of CED guide screw on the mouse skull with distances from the left ear and eye and the simultaneous inhalation anesthesia of three mice, respectively. (**B**) Device ii: the RS 2000 Biological Irradiator (Rad Source Technologies, Alpharetta, GA, USA) equipped with the inhalation anesthesia device. (**C**) Device iii: the irradiation chamber with inhalation anesthesia tubing. (**D**) Device iv: the X-ray collimator with positioning laser beams. (**E**) Dose calibration i: the mouse polystyrene support with RadCal X-ray calibrating probe. (**F**) Dose calibration ii: the RadCal dosimeter display. (**G**) Dose calibration iii: radiochromic films placed under and over the mouse body and positioning the X-rays beam to the brain by the collimator laser. (**H**) Dose calibration iv: the upper radiochromic film before irradiation. (**I**) Dose calibration v- the upper radiochromic film after irradiation. The irradiated region of top head is indicated by the darkened region of the radiochromic film. (**J**) Dose calibration vi: example of dose distribution quantification after scanning of radiochromic film. (**K**) Dose calibration vii: the irradiated region under the head as indicated by the darkened region of the bottom radiochromic film. (**L**) Dose calibration viii: the 70°-rotated animal allowing direct irradiation of the left, tumor-bearing brain hemisphere. (**M**) Dose calibration ix: as in K but with high-sensitivity radiochromic film allowing visualization of the guide screw position (indicated with arrow). (**N**) Dose calibration x: magnification of L with site of guide screw indicated with arrow. (**O**) Dose calibration xi: example of RS 2000 parameters for irradiation with 0.5 Gy to the head and virtually 0.0 Gy to the body.

Mice were provided by the Breeding Unit of the Animal Facility at IRCCS Ospedale Policlinico S.Martino - Genova, Italy and by Envigo (San Pietro al Natisone, Udine, Italy). Immunodeficient non-obese diabetic/severe combined immunodeficient (NOD-SCID) mice were housed in dedicated premises of the animal facility under maximum barrier conditions, one animal per cage to facilitate health status inspection. Barrier conditions included: use of microisolator (filter bonneted) cages; sterilized or disinfected food, water, bedding, cages and anything that come in contact with the mice; only personnel involved in care of the mice had access to the mouse rooms and caretakers wore personal, protective equipment at all times; cages were changed under a laminar flow hood weekly. Immunocompetent mice were housed under ordinary pathogen-free conditions, one animal/cage. Husbandry conditions included a 12 hours light/dark cycle and ad libitum access to standard laboratory chow and water. The experimental unit was the single animal that was uniquely identified by a number marked on the tail and coded by ear punches. This mouse ID number was in the format XX.XX where the first two digits indicate the experiment and the second two digits indicate the animal. In radiosensitization experiments, twenty mice were randomly assigned to the experimental (ten animals) and control (ten animals) groups: unless otherwise indicated, mice in the experimental group were i.c. infused with 12.5 µl of 250 µM KU60019 in 2.5% ethanol/0.9% NaCl. Animals in the control groups were i.c. infused with an equal volume of vehicle. Considering an average mouse brain volume of 415 µl^18^, the brain ethanol concentration after CED was 0.075% that does not cause any serious health effect (limited euphoric status at most)^12^. All other procedures (e.g. radiotherapeutic treatments – Fig. 1) were identical and concomitant for the two groups. The order in which the animals in the experimental and control groups were treated, as well as the animal sex, were alternate in subsequent experiments. Animals were monitored daily in blind by experienced researchers assigning to each animal a health score (H1 moribund status – H10 full health) based on neurological symptoms including posture changes, arched back, tail weakness, diminished activity and skin turgor. Euthanasia was performed by CO_2_ asphyxiation at the moribund status H1 that was the primary experimental outcome in this study.

### Pharmacokinetics of KU60019 after intraperitoneal (i.p.) delivery

Tumor-free immunocompetent C57/Black mice were inoculated i.p. daily on weekdays for two consecutive weeks (total of 10 i.p. injections) with 30 µl/g body weight of vehicle (2.5% ethanol in 0.9% NaCl) or 250 µM KU60019. 16 hours after the last administration, blood was collected by retro-orbital bleeding, the mice were euthanized by CO_2_ asphyxiation and their brains explanted. Blood and brain tissues were immediately extracted for KU60019 content determination as described^13^. Briefly, blood and brain samples were suspended in water and homogenized for 30 sec using an Ystral X1020 homogenizer (Ystral GMBH, Dottingen, Germany). An equal volume of pure methanol was then added, the samples were homogenized for further 60 sec, centrifuged and their supernatants were stored at −80 °C. KU60019 content was determined by High Performance Liquid Chromatography (HPLC)/mass spectrometry (MS) using an Agilent 1200 series chromatographic system (Agilent Technologies, Palo Alto, CA, USA). 10 µl of filtrated sample were injected onto a Symmetry 300 C18 column (Waters Corp., Milford, MA, USA). After separation, the eluent flow was directly sent to an Agilent 6210 TOF mass spectrometer equipped with an electrospray (ESI) ion source operating in positive polarity (Agilent Technologies). High-resolution spectra were recorded in the range m/z 100–1000. The Mass Hunter acquisition software ver.A.02.02 (Agilent Technologies, Palo Alto, CA, USA) was autocalibrating, continuously recording the results of internal reference masses along with the raw data, thereby allowing accurate mass correction. The full-scan data recorded were processed by using Mass Hunter Qualitative Analysis ver.B.02.00 (Agilent Technologies, Palo Alto, CA, USA) and the relative amounts of KU60019 were measured by evaluating the peak areas of the extracted ion currents (EIC m/z 548.22 [M+H]+).

### The adult GIC line COMI

The primary GIC line COMI derived from an adult GB has been previously described^10,12,13,19^ (Fig. 3E–G). Its authentication was performed by determining the proliferation rate, the expression of DDR, stem, *PI3K/Akt* pathway genes as well as the *IDH1*, *TP53*, *H3F3A*, *PDGFRA*, *CDKN2A* and *EGFR* status as previously described^10,12,13,19^. COMI GICs have wild type *TP53* sequence but they express the TP53 RNA at low levels^12^. COMI GICs initiate orthotopic glioma development with >95% efficiency when injected i.c. into immunodeficient NOD-SCID mice^12,13,19^ (Fig. 3H–K). To this aim, 4–5 weeks old NOD/SCID mice were anesthetized with i.m. ketamine and xylazine. Thereafter, the animals were positioned into a stereotaxic frame (David Kopf instruments) and a hole was made, using a 21-gauge needle, 2.5 mm lateral and 1 mm anterior from the intersection of the coronal and sagittal sutures (Bregma). 2–5 × 10^5^ GIC were injected at a depth of 3 mm in correspondence of the left corpus striatum. A guide screw with a central hole fitting a 26 gauge needle (PlasticsOne, Inc, Roanoke, VA) was then inserted into the skull hole to facilitate subsequent drug infusion via convection enhanced delivery [(CED) – drug delivery performed through a catheter connected to an electricity-operated pump] and the skin closed using metal staples (Martin GMBH, Tuttingen, Germany). At least two weeks were left elapsing before CED in order to achieve appropriate guide screw stabilization into the skull bone. In order to avoid significant subpopulation selection during prolonged cell culture, GIC samples frozen after no more than 30 days of culture were used for orthotopic tumor development.

### The pediatric GIC line 239/12

The pediatric GIC line 239/12 was obtained from surgical resection of a left temporo-parietal anaplastic astrocytoma (AA-WHO grade III) in a 14-year-old male. Pediatric GIC were cultured and characterized under the same conditions used for adult COMI GIC. 239/12 GICs expressed glial fibrillary acidic protein (GFAP), vimentin and nestin by immunohistochemistry. Mutation analysis by direct-sequencing and multiplex ligation-dependent probe amplification demonstrated in 239/12 the presence of a frameshift mutation in the *TP53* tumor suppressor gene causing early termination and loss of function of the protein (c.819_820insT; p.V274Cfs*. Reference sequence NM000546 – Supplementary Fig. 1).

### CED, RT, MRI, histology

Intracranial (i.c.) KU60019 administrations to the tumor site were carried out in the surgery room by convection enhanced delivery (CED) as previously described^12,13^ (Fig. 1A). Briefly, at the indicated day after tumor implant, a mid-sagittal skin incision was made on the head and the guide screw exposed. 12.5 µl of KU60019 solution in 2.5% ethanol/0.9%NaCl or vehicle were directly infused into the brain via a cannula inserted into the guide-screw using a BeeHive electrically-motorized pump (BeeHive™, Bioanalytical Systems, West Lafayette, Indiana) set at rate of 0.5 µl/minute. This procedure did not cause any evident interference with the cognitive behavior of the mice nor increased susceptibility to bacterial infections, suggesting absence of effects on the immune system.

RT of orthotopic GB was performed by an RS 2000 Biological Irradiator (Rad Source Technologies, Alpharetta, GA, USA - Fig. 1B) whose “*in vivo*” delivered dose was verified by a RadCal Accu-Gold system (Monrovia, CA, USA) equipped with a 10 × 6–0.6 High Dose Rate Chamber (Fig. 1E,F). The dose was confirmed by two radiochromic films (Gafchromic® EBT3, Ashland Inc., Covington, KY, USA) placed over and under the mouse body (Fig. 1G–O). The prescription dose was 0.5 or 2.5 Gy. In some experiments, RT was also performed on the head of mice by a HDR (high dose rate) brachytherapy delivery system (microSelectron Digital, Nucletron Elekta, Stockholm, Sweden) as previously described^13^. Radiation toxicity studies were performed on both immune/repair-deficient NOD-SCID mice bearing adult orthotopic tumors and immune/repair-competent tumor-free wild type C57/Black mice (Fig. 4). In order to reduce toxicity of anesthesia during repeated drug administrations and RT, an isoflurane inhalation anesthesia apparatus was used including: an Isoflurane Vaporizer (Rothacher-Medical, CH-3000, Berne Switzerland); a 2B LFY-I-5A Medical Oxygen Concentrator (2Biol, Besozzo, Italy); an anesthesia induction chamber (E-Z Anesthesia, E-Z Systems Corporation, Palmer, PA) and a dispersed isoflurane captation device (Fluovac Harvard Apparatus with Veterinary Fluosorber) (Fig. 1B–D).

MRI of the orthotopic tumors was performed under ketamine-xylazine i.p. anesthesia by a clinical 3 T whole body scanner (Signa EXCITE®HDxT, GE, Milwaukee, USA) with mice positioned in a prototype coil (linear birdcage transmit/receive coil, Flick Engineering Solutions BV-General Electric) as described^12^. Tumor volumes were considered as secondary experimental outcomes. They were determined by Growing Region Segmentation Software (GRES) for quantitative magnetic resonance imaging as described^20^. Briefly, the software determines the tumor area (segmentation) in any single slice and the tumor volume is calculated multiplying the areas for the slice thickness (1 mm). The region grows as a frame starting from a seed identified by the operator, including pixels that are spatially connected and have their signal level above a threshold. The procedure ends when the latter conditions are no more satisfied.

For histological analysis, animals were euthanized by CO_2_ asphyxiation and brains were cryopreserved. Coronal sections obtained at a cryostat microtome were fixed and stained with hematoxylin/eosin (H/E) or with an anti-human nestin mouse monoclonal primary antibody (1 µg/ml - Abcam, Cambridge, UK) followed by a FITC-conjugated goat anti-mouse secondary IgG and DAPI counterstaining.

### Hematological analyses and ATM inhibition

For hematological analyses (Fig. 4D,E), 0.5–0.7 ml of blood was obtained from C57 Black mice by retro-orbital withdrawal in lithium-heparinized vessels without separating gel. Samples were then analyzed by a Sysmex sp-1000i automated slide preparer-stainer (Sysmex Corporation, Kobe, Japan) equipped with CellaVision® DM-96 morphological cell analyzer (CellaVision AB Lund, Sweden).

The capacity of the KU60019 preparations to inhibit ATM kinase *in vivo* was determined by (a) immunofluorescence (IF – Fig. 5A,B): mice bearing orthotopic adult COMI GB developed for 52 days were inoculated by CED at the tumor site with 12.5 µl of 250 µM KU60019 or vehicle (12.5 µl of 2.5% ethanol in 0.9% NaCl) followed by irradiation to the head with 2.5 Gy at d56, 57 and 58. The radiosensitization cycle was then repeated with CED at d59 followed by 2.5 Gy at d63 and d64 (total dose delivered: 12.5 Gy). At d65, moribund animals were euthanized by CO_2_ asphyxiation, the brains removed and slices obtained by cryostat sectioning. The orthotopic tumor tissue was fixed, permeabilized, blocked with normal goat serum and incubated with rabbit monoclonal antibody to ATM-phospho S1981 (1:500 - Abcam, Cambridge, UK) followed by fluorochrome-conjugated antibody (goat anti-rabbit Alexa Fluor-488, Molecular Probes, Oregon, USA). After counterstaining with DAPI (Sigma-Aldrich, Milano, Italy), nuclei were scored at 400X magnification and considered positive when containing 3 or more ATM-phospho S1981 foci. The percentage of ATM-phospho S1981-positive cells was calculated after counting in blind at least 740 cells in 45 randomly selected fields. Images were always taken under equal conditions of brightness and contrast. (b) enhancement of KI67 immunostaining in orthotopic tumors specimens, as described (Fig. 5C,D)^13^: briefly, mice bearing orthotopic COMI GB developed for 52 days were inoculated by CED at the tumor site with 20 µl of 250 µM KU60019 or vehicle (20 µl of 2.5% ethanol in 0.9% NaCl) followed by irradiation with 0.5 Gy at d56, 57 and 58. The radiosensitization cycle was then repeated with CED at d59 followed by 1.5 Gy at d63 and with CED at d66 followed by 1.5 Gy at d70, 71 and 72 (total dose delivered: 7.5 Gy). At the indicated times [(d); Fig. 5C], animals were euthanized by CO_2_ asphyxiation, the brains removed and fixed in 10% formaldehyde. Sections of the brain were stained with hematoxylin/eosin (H/E) and the orthotopic tumor tissue subjected to immunohistochemistry with anti-KI67 antibody (5 µg/L - Ventana-Roche, Tucson, AZ, USA). The percentage of KI67-positive cells was evaluated at 200X magnification counting in blind at least 1350 cells in 30 randomly selected fields.

### Statistical analysis

Ten mice per treatment group were used. This number was calculated *a priori* by the Gpower software (http://www.gpower.hhu.de/) based on an alpha error probability value of 0.05 for a 10% difference of median survivals in the experimental and control groups and taking into account occasional losses of animals unrelated to tumor progression (e.g. during CED - Figs 4A, 6A,E and 7Q). Percentages of ATM-phospho S1981- and KI67-postive cells were compared by one-way ANOVA with Tukey’s Multiple Comparison test after counting in blind at least 740 cells in 45 randomly selected fields and 1350 cells in 30 randomly selected fields, respectively (Fig. 5B,D). Tumor volumes as determined by the GRES method (Fig. 6D) were compared by two-tailed unpaired t-test. The GraphPad Prism 5.01 statistical software was used.

## Results

### KU60019 does not cross the blood brain barrier (BBB)

**Figure 2:**
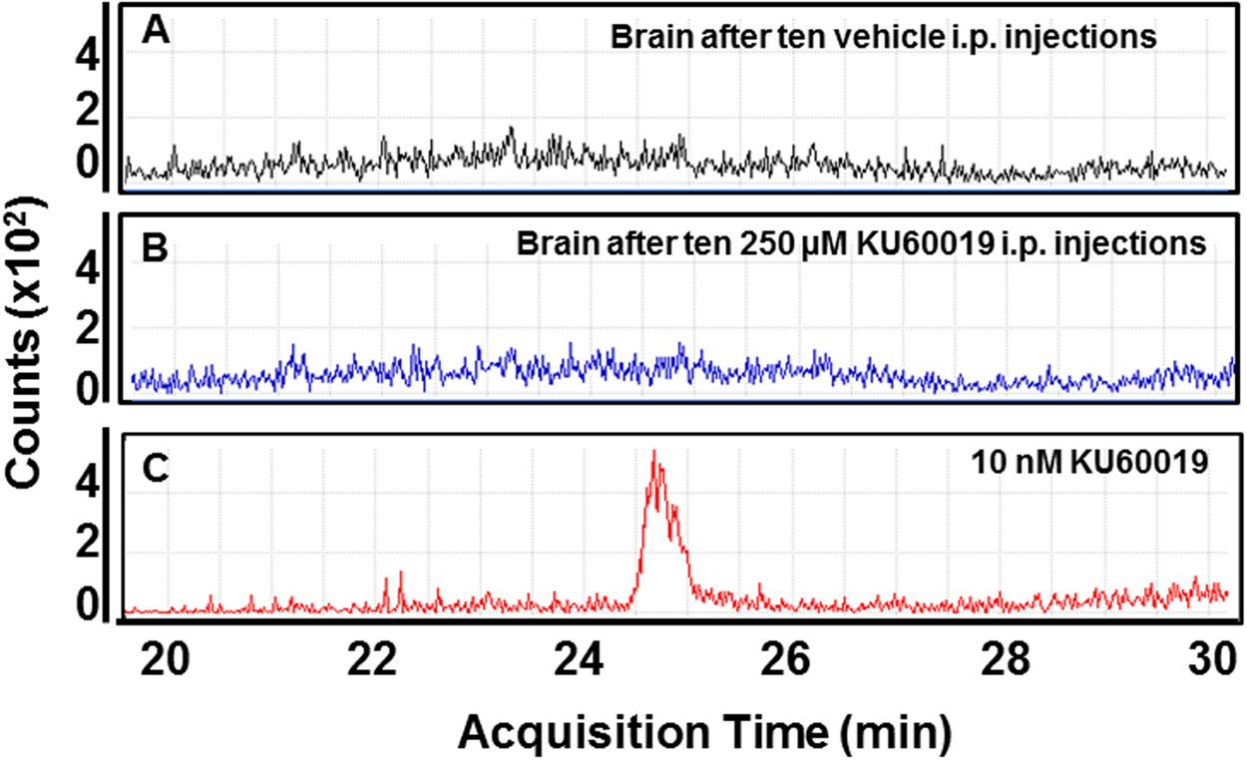
Inability of KU60019 to cross the BBB after chronic administration. Healthy C57/Black mice were inoculated i.p. daily during two consecutive weeks (total of 10 injections) with 30 µl/g body weight of (**A**) vehicle (2.5% ethanol in 0.9% NaCl) or (**B**) 250 µM KU60019. Mice were then euthanized by CO_2_ asphyxiation, the brain removed and whole brain extracts prepared as described in Materials and Methods and^13^. KU60019 content was determined by HPLC/MS as described in Materials and Methods and^13^. KU60019 amounts higher than limit of quantitation (LoQ - 5 nM) were never found in brain tissues. **C**. As a chromatographic marker, 10 nM KU60019 was run under the same conditions.

**Figure 3:**
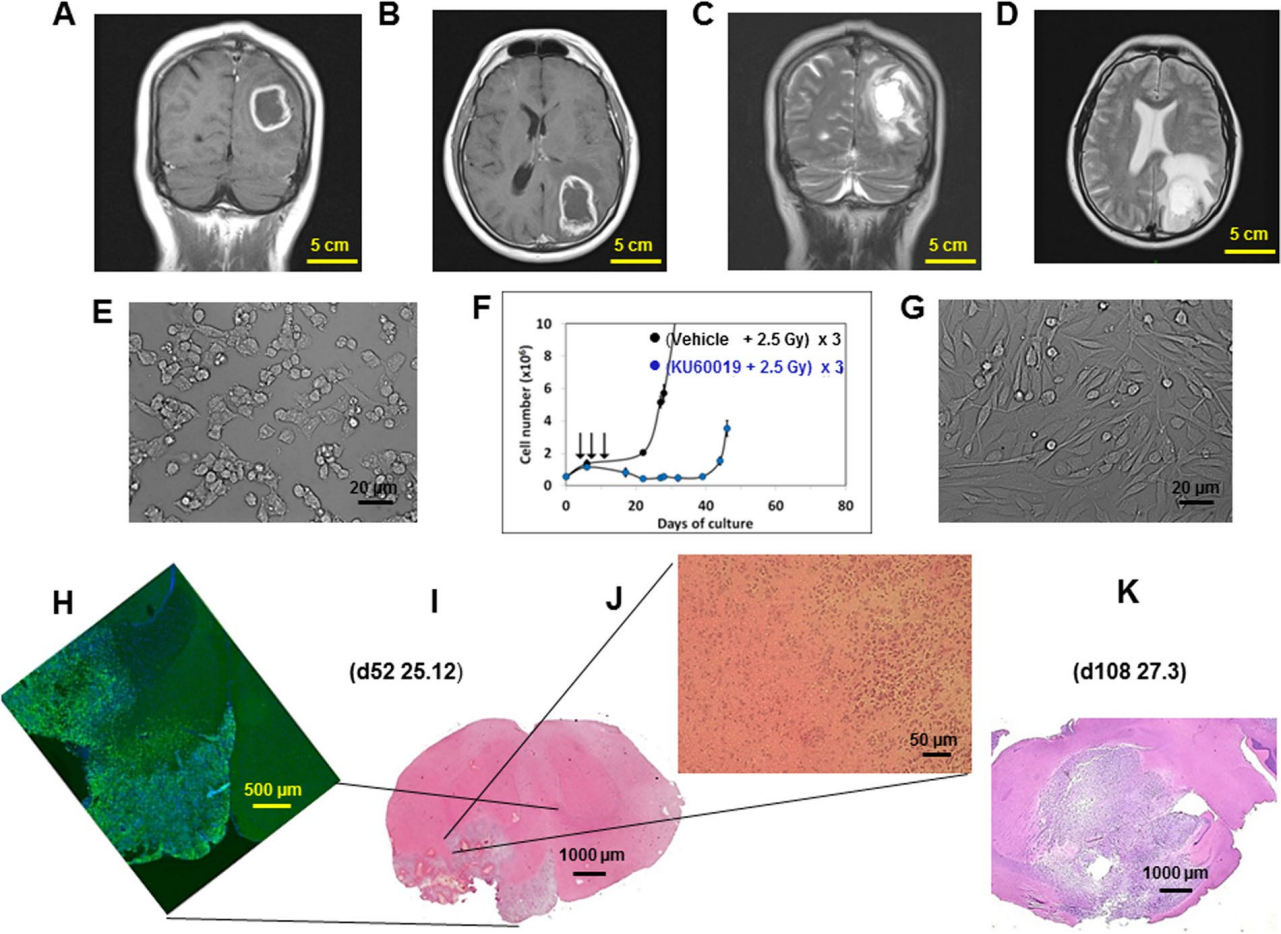
The GIC-driven COMI tumor. (**A**,**B**) Patient’s tumor i: coronal and axial T1-weighted post-contrast MRI showing a left parieto-occipital intra-axial tumor with irregular contrast enhancement and necrotic core. (**C**,**D**) Patient’s tumor ii: T2-weighted images showing significant peritumoral edema and infiltrative behavior. (**E**) GIC i: the GIC component of the tumor was isolated immediately after surgery. Under matrigel-coating and serum-free conditions, the cells grew and layered onto a monolayer, maintaining intact self-renewal capacity. (**F**) GIC ii: the COMI GIC could be effectively radiosensitized *in vitro* by exposure to 1 µM KU60019 30 min prior to irradiation with three 2.5 Gy IR fractions [reproduced from^12^, with permission]. (**G**) GIC iii: removal of growth factors and addition of 10% FCS to the proliferation medium after approximately two weeks induced GIC differentiation with acquisition of astrocytic morphology and altered refractory index. (**H**–**K**) Orthotopic tumor. 3 × 10^5^ COMI serum-free grown GIC were stereotactically injected in the left corpus striatum of immunodeficient NOD SCID mice. Staining with hematoxylin/eosin of brain tissue sections revealed 52 (**I**,**J**) and 108 (**K**) days later a growth pattern of the orthotopic tumor reflecting the characteristics of the clinical tumor: a voluminous expansive lesion (**I**,**K**) with an irregular infiltrating wall (**J**) exerting mass effect on the adjacent structures (**K**). Immunostaining at d52 revealed that most of the orthotopic tumor expressed the stem cell marker nestin, indicating its stem cell-driven character (**H**). Mouse ID numbers 25.12 and 27.3 are indicated for the sake of reference with the days (d) of tumor development.

Previous *in vitro* work in our laboratory on cultured GIC has shown that an increasing number of radiosensitizing cycles each consisting of exposure of cells to 1 µM KU60019 followed 30 min later by irradiation with 2.5 Gy could substantially delay (Figs 3F and 7J) and even eliminate the recovery of radioresistant cells, as compared to irradiated GIC exposed to vehicle only^12^. In order to translate this radiosensitizing effect to the animal setting, we had also investigated the pharmacokinetics of KU60019 in tumor-free mice finding that, unlike blood, liver and kidneys, no drug could be detected in the brain parenchyma after one i.p. administration, indicating that KU60019 could not cross the BBB^13^. However, since traces of drug close to the limit of quantitation could be detected in the brain parenchyma by HPLC/MS and by virtue of the established low/null toxicity of KU60019^12^, we wished to investigate whether the chronic i.p administration may produce a KU60019 accumulation in the brain, reaching a concentration sufficient to radiosensitize the orthotopic tumors (Fig. 2). Ten i.p injections of 250 µM KU60019 at 30 µl/g body weight were carried out in tumor-free mice every weekday during two consecutive weeks (Fig. 2B). In agreement with previous data, no significant signs of toxicity were observed^12,13^. 16 hours after the last i.p. injection the animals were euthanized, the brains explanted and the content of KU60019 in brain extracts measured by HPLC/MS as described under Materials and Methods and in^13^. No significant amounts of KU60019 were detected in the brain of animals even under those conditions of chronic administration (Fig. 2B), confirming the impermeability of the BBB to KU60019.

### The GIC-driven adult COMI tumor

Biddlestone-Thorpe and coworkers have reported that ATM kinase inhibition by KU60019 preferentially sensitizes *TP53* mutant orthotopic gliomas to IR^17^. In that study, 250 µM KU60019 was i.c.- delivered by osmotic pumps or CED to orthotopic tumors driven by the established U1242 and U87 cell lines carrying the R175H and D281G *TP53* mutations, respectively. Here we wished to investigate whether KU60019 displayed similar radiosensitization properties towards GIC-driven orthotopic tumors that might more faithfully mimic the growth patterns of clinical tumors. The selected adult patient was a 48-year-old man who, in full well-being, complained about the onset of two episodes of violent headache in the left parieto-occipital region which resolved spontaneously. In the following days his relatives noticed a progressive space-time disorientation so that, after neurological examination, the patient was subjected to brain MRI which showed the presence of a voluminous expansive lesion in the left temporo-parieto-occipital site, associated with perilesional edema, with a central necrotic-colliquative component and an irregular wall that showed remarkable and inhomogeneous intake of contrast medium (Fig. 3A–D). This lesion, compatible in the first instance with a HGG lesion, exerted considerable mass effect on the adjacent structures (Fig. 3B,D). The patient was subjected to surgery to remove the lesion and histological examination of the tumor led to diagnosis of GB (WHO grade IV). Despite postoperative chemo- and radiotherapy the patient eventually died 413 days after diagnosis. Immediately after surgery, tissue surplus to diagnostic requirements was processed for isolation of GIC, according to^10^. Under matrigel-coating and serum-free conditions, the cells grew and layered into a monolayer (Fig. 3E), maintaining intact self-renewal capacity. In the absence of matrigel, cells predominantly grew forming suspended neurospheres. The COMI GIC could be effectively dislodged from their quiescence (population doubling time decreased from 60 to 49 hours) and radiosensitized, after exposure to 1 µM KU60019 30 min prior to irradiation with three 2.5 Gy IR fractions (Fig. 3F)^12^. Removal of growth factors and addition of 10% FCS to the proliferation medium, after approximately two weeks induced GIC differentiation with acquisition of astrocytic morphology, altered refractory index (Fig. 3G)^12^, increased expression of a number of differentiation markers including GFAP and beta III tubulin and refractoriness to radiosensitization by KU60019^10,19^. 3 × 10^5^ COMI serum-free grown GIC were then stereotactically injected into the left corpus striatum of NOD SCID mice. Staining with hematoxylin/eosin of brain tissue sections revealed 52 (Fig. 3I,J) and 108 (Fig. 3K) days later a growth pattern of the orthotopic tumor reflecting the characteristics of the clinical tumor: a voluminous expansive lesion (Fig. 3I,K) with an irregular infiltrating wall (Fig. 3J) that exerted considerable mass effect on the adjacent structures (Fig. 3K). Immunostaining at d52 revealed that most of the orthotopic tumor expressed the stem cell marker nestin, indicating its stem cell-driven character (Fig. 3H).

### CED and RT of GIC-driven orthotopic tumors

Due to the impermeability of the BBB to KU60019 (Fig. 2) and based on previous data, in order to effectively radiosensitize the brain tumor, KU60019 must be delivered i.c. and multiple radiosensitization cycles have to be carried out^12,13,17^. To this aim and in accordance with the principle of 3Rs (replacement, refinement, reduction) in animal research, two major technical improvements were adopted. First, since animals would not survive to frequent systemic ketamine-xylazine anesthesia, a less toxic isoflurane (inhalation anesthetic) delivery system was employed (Fig. 1A,B). Second, one on-site dedicated animal irradiator equipped with a collimator directing the radiation beam to the brain only was calibrated and employed (Fig. 1B–O).

**Figure 4:**
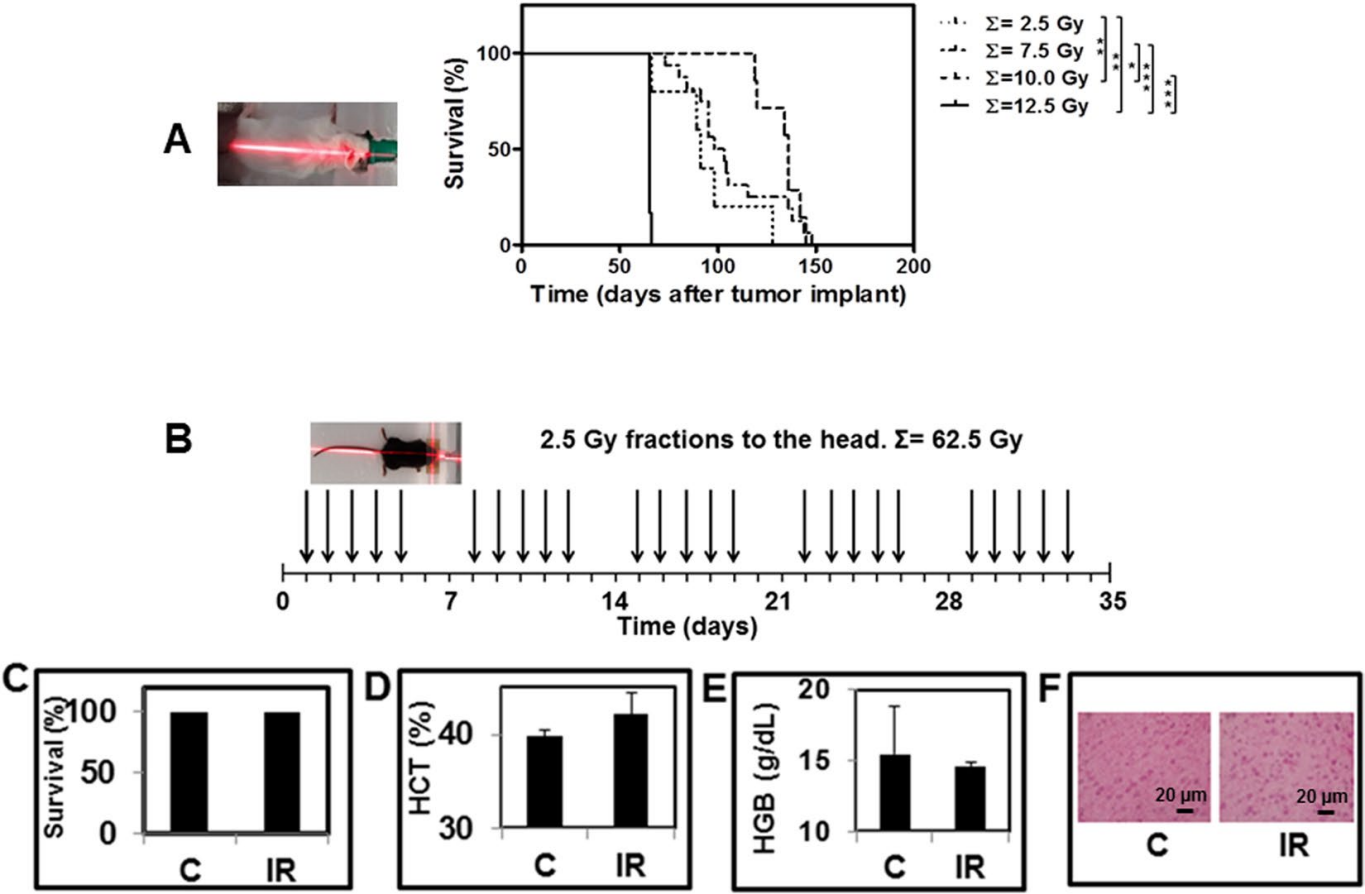
RT of GIC-driven orthotopic tumors. (**A**) 4–5 weeks old NOD-SCID mice were stereotactically injected in the left corpus striatum with 2–5 × 10^5^ COMI GIC. 50 days later, mice were i.c.-administered with vehicle (2.5% ethanol in 0.9% NaCl) and then irradiated with one (total: 2.5 Gy), three (total: 7.5 Gy), four (total: 10.0 Gy) or five (total: 12.5 Gy) 2.5 Gy fractions to the head. Increased survival was observed up to the 10.0 Gy cumulative IR dose. Additional 2.5 Gy (cumulating to a total of 12. 5 Gy) were rapidly lethal with all mice dying in 24–48 hours (solid line). Differences between survival curves with P < 0.05, 0.01, 0.001 are indicated with one, two and three asterisks, respectively. (**B**) For comparison, the heads of tumor-free wild type C57Black mice (left) were irradiated every weekday with 2.5 Gy over five weeks (total dose delivered: 62.5 Gy). Blood samples were then drawn for hematology and the brains explanted for histopathology. No significant variation of (**C**) animal survival; (**D**) hematocrit; (**E**) hemoglobin; (**F**) normal brain histology was observed in irradiated (IR) versus unirradiated (**C**) mice.

The maximum deliverable radiation dose to the mice bearing the COMI tumor was preliminarily determined (Fig. 4A). In radiosensitive NOD-SCID mice bearing orthotopic adult COMI tumors, a dose – therapy relationship was observed in the range 0–10 Gy, delivered by 2.5 Gy fractions to the head every 24 hours (2.5 vs 10 Gy, 91 vs 136 days of median survival, ratio: 0.669, 95% CI of ratio: 0.351 to 0.986, P: 0.0040 indicated by two asterisks; 7.5 vs 10 Gy, 100.5 vs 136 days of median survival, ratio: 0.739, 95% CI of ratio: 0.328 to 1.150, P: 0.0310 indicated by one asterisk; Fig. 4A, right). After addition of one fifth 2.5 Gy fraction [bringing the total cumulative (∑=) dose to 12.5 Gy] all animals rapidly died (in 24–48 hours), indicating that a cumulative radiation dose higher than 10 Gy to the head is invariably lethal to the DNA repair–deficient NOD-SCID mice (10 vs 12.5 Gy, 136 vs 65 days of median survival, ratio: 2.092, 95% CI of ratio: 1.756 to 2.428, P: 0.0005 indicated by three asterisks; 7.5 vs 12.5 Gy, 100.5 vs 65 days of median survival, ratio: 1.546, 95% CI of ratio: 1.155 to 1.937, P < 0.0001 indicated by three asterisks; 2.5 vs 12.5 Gy, 91 vs 65 days of median survival, ratio: 1.400, 95% CI of ratio: 1.095 to 1.705, P:0.0034 indicated by two asterisks; Fig. 4A, right). The multiple defects in both innate and adaptive immunity in NOD-SCID mice are necessary in order to achieve reproducible engraftment of GIC-driven orthotopic tumors in virtually 100% of mice^21^. For comparison and in the view of the possible clinical translation of these studies, we wished to deepen our understanding of radiation toxicity to the head of wild type DNA repair competent C57/Black mice (Fig. 4B). No significant variation of survival (Fig. 4C), hematocrit (HCT-Fig. 4D), hemoglobin (HGB-Fig. 4E) or brain histology (Fig. 4F) were observed after twenty-five 2.5 Gy fractions to the head each other 24 hours apart (cumulative radiation dose: 62.5 Gy) (Fig. 4B), thus showing the feasibility of an elevated number of radiosensitization cycles in DNA repair competent organisms such as GB patients usually are.

### KU60019 radiosensitizes GIC-driven adult orthotopic tumors and improves animal survival

**Figure 5:**
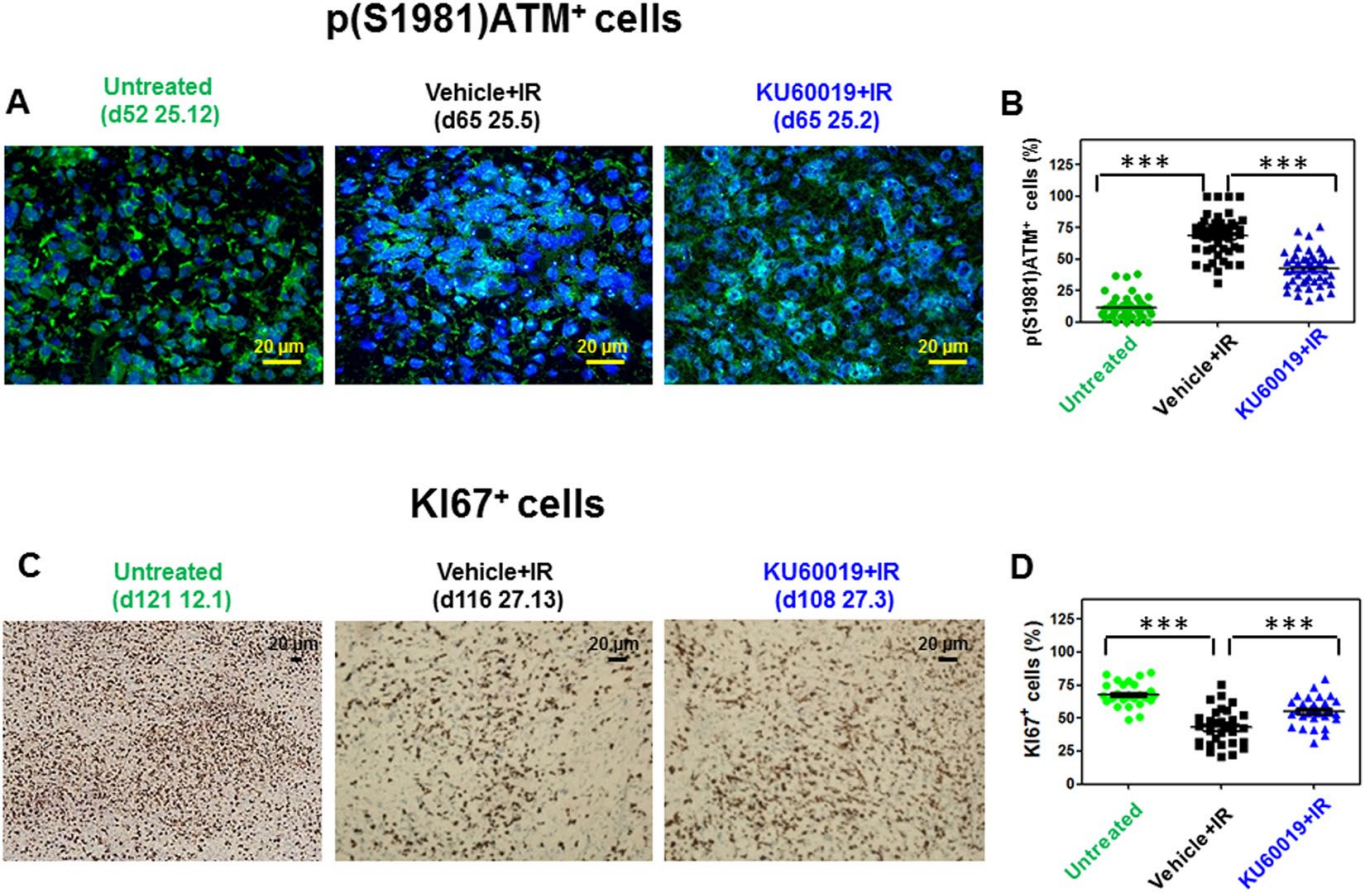
*In vivo* ATM inhibition by KU60019. (**A**) Immunodeficient mice bearing orthotopic COMI GB developed for 52 days were inoculated by CED at the tumor site with 12.5 µl of 250 µM KU60019 or vehicle (2.5% ethanol in 0.9% NaCl) followed by irradiation with 2.5 Gy at d56, 57 and 58. The radiosensitization cycle was then repeated with CED at d59 followed by 2.5 Gy at d63 and d64 (total dose delivered: 12.5 Gy). At d65, moribund animals were euthanized by CO_2_ asphyxiation, the brains removed and slices obtained by cryostat sectioning. The orthotopic tumor tissue was processed for IF analysis with rabbit monoclonal antibody to ATM-phospho S1981 as described under Materials and Methods. Reduced pATM foci in [KU60019 + IR (2.5 Gy × 5)] as compared to [Vehicle + IR (2.5 Gy x 5)] -treated tumors is shown. Mouse ID numbers 25.12, 25.5 and 25.2 are indicated for the sake of reference with the days (d) of tumor development. (**B**) The frequency distributions of the percentages of ATM-phospho S1981-positive cells in 45 randomly selected fields are shown. The three asterisks indicate P < 0.0001 for the differences of the frequency distributions between the indicated groups as determined by ANOVA (25.5 vs 25.12 mean difference +57% of p(S1981)ATM^+^ cells, 95% CI of difference +50 to +64%; 25.2 vs 25.5 mean difference −26% of p(S1981)ATM^+^ cells, 95% CI of difference −19 to −33%; 25.12 vs 25.2 mean difference −31% of p(S1981) ATM+ cells, 95% CI of difference −24 to −38%). (**C**) Persistent increase of KI67 staining in KU60019-treated mice 42 days after CED. At d52 of tumor development, COMI tumor-bearing mice were i.c.- injected by CED with 20 µl of 250 µM KU60019 or vehicle (2.5% ethanol in 0.9% NaCl) followed by irradiation with 0.5 Gy at d56, 57 and 58. The radiosensitization cycle was then repeated with CED at d59 followed by 1.5 Gy at d63 and with CED at d66 followed by 1.5 Gy at d70, 71 and 72 (total dose delivered: 7.5 Gy) Mouse ID numbers 12.1, 27.13 and 27.3 are indicated for the sake of reference with the days (d) of tumor development. (**D**) The frequency distributions of the percentages of KI67-positive cells in 30 randomly selected fields are shown. The three asterisks indicate P < 0.0001 for the differences of the frequency distributions between the indicated groups as determined by ANOVA (27.13 vs 12.1 mean difference −25% of KI67^+^ cells, 95% CI of difference −18 to −32%; 27.3 vs 27.13 mean difference +12% of KI67+ cells, 95% CI of difference +5 to +19%; 12.1 vs 27.3 mean difference +13% of KI67+ cells, 95% CI of difference +6 to +19%).

The (12,5 µl of 250 µM) dose employed by Biddlestone-Thorpe and coworkers was confirmed in our studies to be effective in inhibiting the ATM protein with consequent release of ATM-mediated checkpoints and induction of the KI67 proliferative marker in the GIC-driven orthotopic tumors^10,12,13^. Figure 5 shows the inhibition of ATM protein by KU60019 in the irradiated *TP53*-deficient COMI adult orthotopic tumors. In Fig. 5A,B COMI tumors were treated with two radiosensitizing cycles, the first consisting of one CED with 12.5 µl of 250 µM KU60019 followed by three 2.5 Gy IR fractions and the second of one CED with the same KU60019 dose followed by two 2.5 Gy IR fractions. Significant activation of ATM protein, as indicated by phosphorylation at S1981 detected through IF foci with a specific antibody, was observed in vehicle + IR - treated mice (Fig. 5A central panel, Fig. 5B black symbols) as compared to untreated mice (Fig. 5A left panel, Fig. 5B green symbols) (25.5 vs 25.12 mean difference: +57% of p(S1981)ATM^+^ cells; 95%; CI of difference +50 to +64%). The p(S1981)ATM foci detected 24 hours after the fifth 2.5 Gy IR fraction were significantly reduced in the KU60019-treated tumors (Fig. 5A right panel, Fig. 5B blue symbols) (25.2 vs 25.5 mean difference: −26% of p(S1981)ATM+ cells; 95% CI of difference −19 to −33%). These results confirm and extend previous evidences of effective ATM inhibition by the employed doses of KU60019 as determined by TP53-phosphorylation assays, radiosensitization of the same GIC lines used for orthotopic tumor development and the induction of the proliferative marker KI67 *in vivo*, consequent to release of the G1/S, intra-S and G2/M checkpoints^10,12,13^. Figure 5C,D show that the elevated proliferative activity in the untreated tumor (Fig. 5C left panel, mouse 12.1, Fig. 5D green symbols) was reduced after RT (Fig. 5C central panel, mouse 27.13, Fig. 5D black symbols) (27.13 vs 12.1 mean difference: −25% of KI67^+^ cells, 95% CI of difference −18 to −32%). KU60019 exposure induced proliferation boosting (Fig. 5C right panel, mouse 27.3, Fig. 5D blue symbols) (27.3 vs 27.13 mean difference: +12% of KI67^+^ cells; 95% CI of difference +5 to +19%). Importantly, the KU60019-mediated stimulus to GIC-mitosis *in vivo* was prolonged in time (Fig. 5C,D), since the significantly increased proliferative activity of GIC, as indicated by KI67 tumor staining, was detected 42 days (d108 of tumor development) after the last drug CED (d66 of tumor development) (Fig. 5C,D).

**Figure 6:**
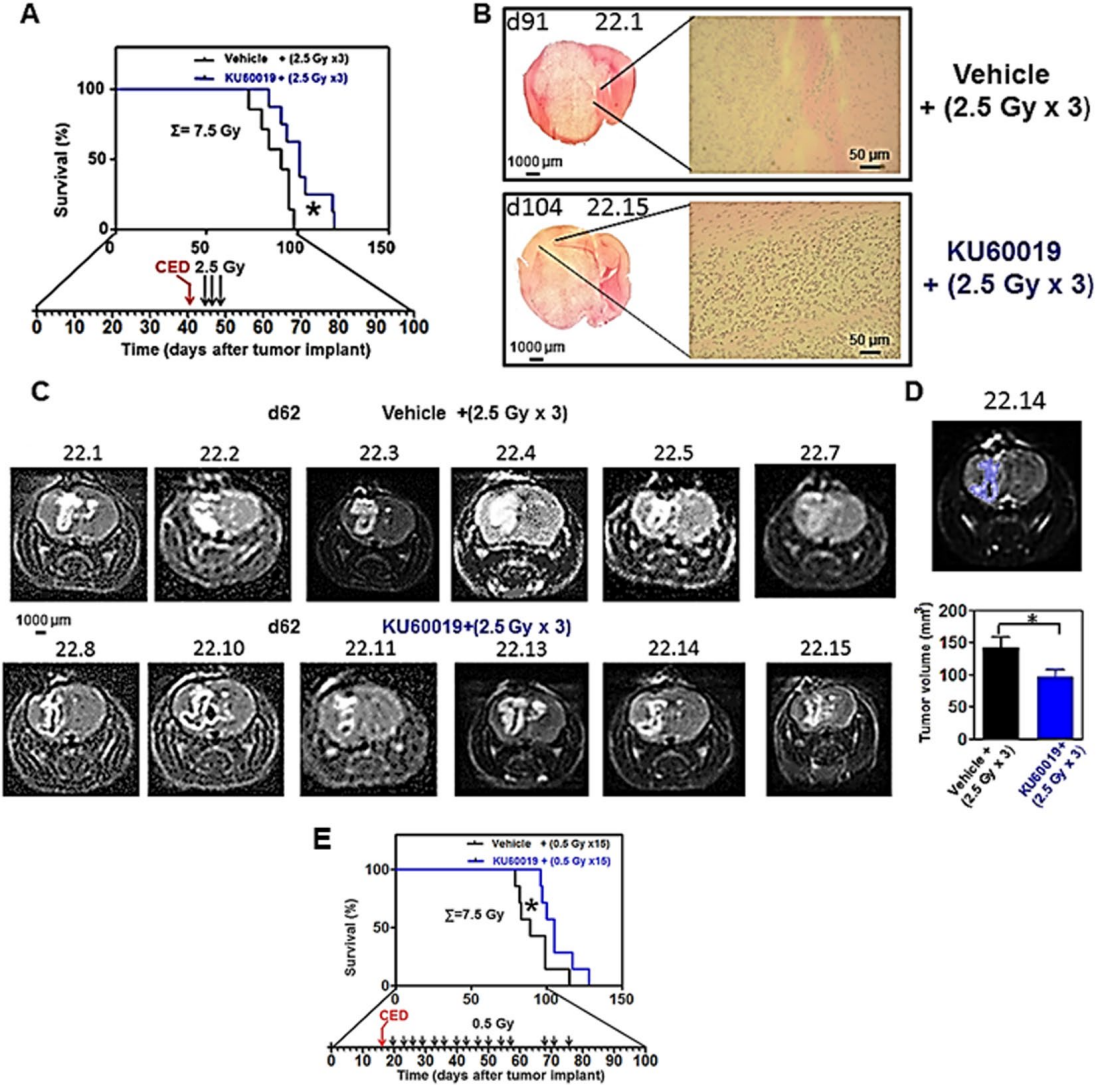
Increased survival of NOD-SCID mice bearing orthotopic adult COMI GB treated by KU60019 plus RT. At d41 of tumor development, animals were i.c.-injected by CED with 12.5 µl of 250 µM KU60019 (blue) or vehicle (ethanol in 0.9% NaCl - black) followed by irradiation with 2.5 Gy to the head at d45, 46 and 48 (total dose delivered: 7.5 Gy). (**A**) Kaplan-Meier survival curves. Median survival of KU60019-treated animals was significantly longer than that of vehicle-treated controls (101 vs 91 days; ratio: 0.901; 95% CI of ratio: 0.5384 to 1.264; P: 0.0409 indicated by one asterisk). (**B**) H/E staining of tumors at euthanasia (d91 and 104 in vehicle and KU60019-injected mice, respectively). Mouse ID numbers 22.1 and 22.15 are indicated for the sake of reference. Left: 2X snap photographs. Right: 100X magnifications showing tumor infiltrating cells. (**C**) 3 T MRI of orthotopic tumors carried out at d62. Top: tumors treated with vehicle+ (2.5 Gy × 3). Bottom: tumors treated with KU60019+ (2.5 Gy × 3). Mouse ID numbers are indicated for the sake of reference. (**D**) Determination of tumor volumes by region segmentation software (GRES). Top: example of segmented tumor. The mouse ID number (22.14) is indicated for the sake of reference. Bottom: mean ± SEM volumes of vehicle + (2.5 Gy × 3) (black) and KU60019+ (2.5 Gy × 3) (blue) – treated tumors. The tumor volumes in KU60019+ (2.5 Gy × 3) – treated mice were significantly reduced as compared to vehicle + (2.5 Gy × 3) -treated mice. Mean ± SEM of vehicle+ (2.5 Gy × 3): 140 ± 17 mm^3^; mean ± SEM of KU60019+ (2.5 Gy × 3): 97 ± 11 mm^3^; P: 0.047 after two-tailed unpaired t-test. (**E**) Further beneficial effect on animal survival after RT hyperfractionation. At d16 of tumor development, animals were i.c.-injected by CED in the surgery room with 12.5 µl of 250 µM KU60019 (blue) or vehicle (ethanol in 0.9% NaCl - black) followed by irradiation with 15 fractions of 0.5 Gy IR delivered to the head of mice 2–4 days apart (total dose delivered: 7.5 Gy). Kaplan-Meier survival curves are shown. Median survival of KU60019-treated animals was further increased (105 vs 89 days; ratio: 0.847; 95% CI of ratio: 0.4969 to 1.198; P: 0.0417) with respect to controls as compared to standard 2.5 Gy fractions as shown in **A**.

Significant elongation of median survival was observed in COMI-bearing animals after one single CED of KU60019 (performed at d41 of tumor development) followed four-seven days later (d45,46,48) by three 2.5 Gy RT fractions as compared to vehicle + IR - treated mice (101 vs 91 days of median survival; ratio: 0.901; 95% CI of ratio: 0.5384 to 1.264; P: 0.0409 indicated by one asterisk; Fig. 6A). Histological analyses performed at euthanasia, showed in both groups large tumor masses whose development was delayed in the KU60019- treated animals (Fig. 6B). 3T MRI imaging performed at d62 and determination of tumor volumes by GRES, consistently showed significant reductions of tumor volumes in radiosensitized mice [mean ± SEM of vehicle + (2.5 Gy × 3): 140 ± 17 mm^3^; mean ± SEM of KU60019 + (2.5 Gy × 3): 97 ± 11 mm^3^; P: 0.047 after two-tailed unpaired t-test indicated by one asterisk (Fig. 6C,D)]^20^. The reduced tumor volumes as assessed by histological analysis and MRI were considered as a secondary experimental outcome in this study. Increasing the radiation dose to total 10 Gy (∑ = 10 Gy) by addition of a fourth 2.5 Gy fraction to mice intracranially infused with KU60019 using osmotic pumps, could not further improve longevity. On the contrary, the beneficial effect observed in Fig. 6A was lost (Supplementary Fig. 2). To better exploit the prolonged proliferation-boosting, radiosensitizing effect of KU60019, an hyperfractionated RT schedule was adopted (Fig. 6E). Tumor bearing mice were i.c.-injected at d16 of tumor development by CED with 12.5 µl of 250 µM KU60019 (blue) or vehicle (2.5% ethanol in 0.9% NaCl - black) followed by irradiation with 15 fractions of 0.5 Gy IR delivered to the head of mice 2–4 days apart from d20 to d76 of tumor development (total dose delivered: 7.5 Gy). Median survival of KU60019-treated animals was further increased with respect to controls as compared to standard fractionation with 2.5 Gy doses in Fig. 6A (105 vs 89 days; ratio: 0.847; 95% CI of ratio 0.4969 to 1.198; P = 0.0417 indicated by one asterisk; Fig. 6E) showing that it is possible to take advantage of the long term proliferation-boosting, radiosensitizing effect of KU60019 over the GIC component of the tumor by adoption of an hyperfractionated RT schedule.

### KU60019 radiosensitizes pediatric GIC-driven orthotopic tumors

239/12 was a 14 years-old boy with a previous unremarkable clinical history who presented at the Emergency Unit of Giannina Gaslini Children’s Hospital because of generalized seizures unresponsive to first line therapy. After CT and MR imaging suggestive of a HGG, biopsy of the lesion led to diagnosis of anaplastic astrocytoma (WHO grade III), positive for GFAP, negative for TP53 overexpression, exhibiting a KI67 proliferation index of 25%. A subtotal resection was thereafter performed. Patient was referred for focal RT and was treated using intensity modulated radiotherapy (IMRT) to a total dose of 60 Gy in 2 Gy daily fractions 5 days per week. Simultaneously, temozolomide chemotherapy was started. The patient died because of progressive disease at d551 after diagnosis.

**Figure 7:**
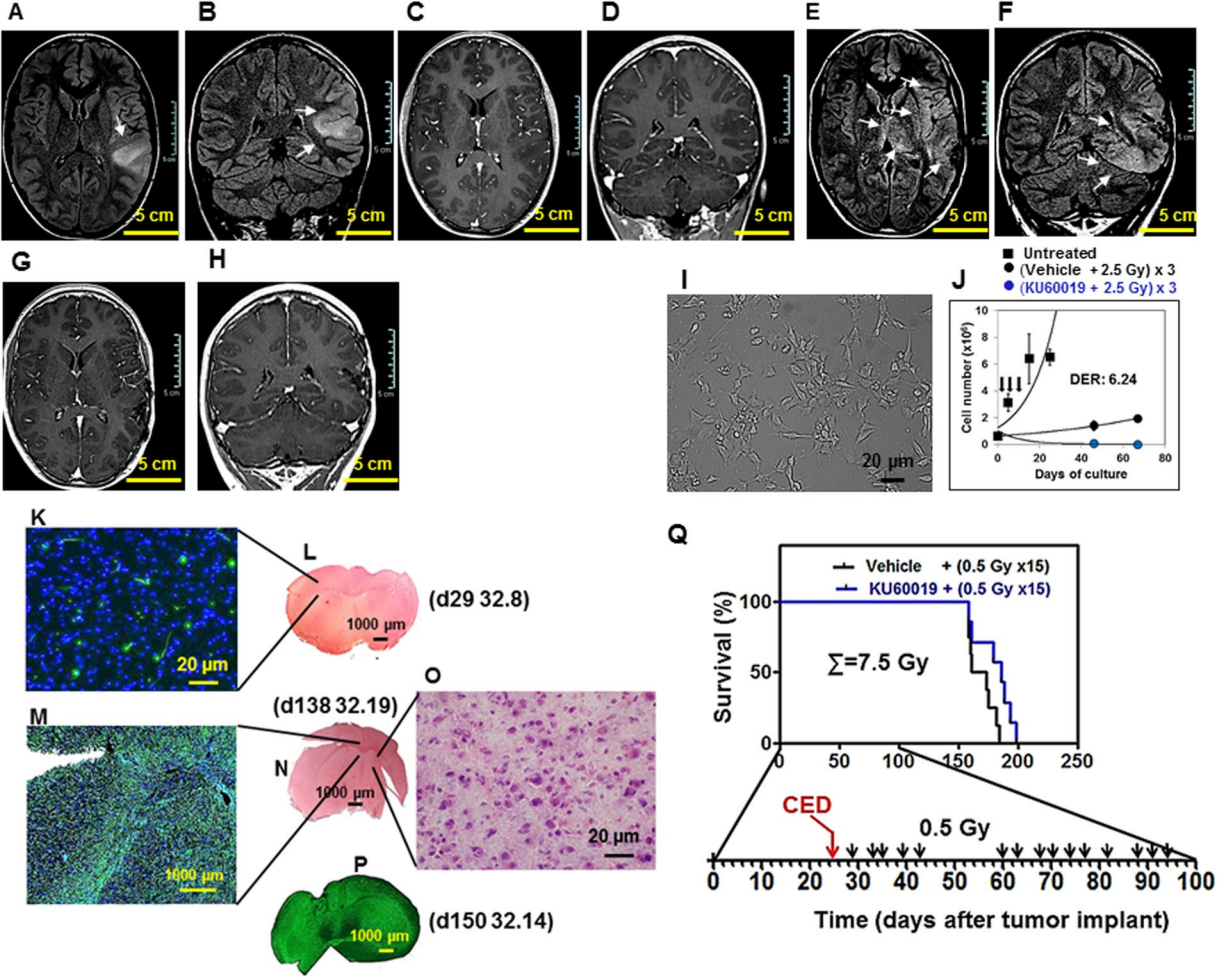
The GIC-driven pediatric 239/12 tumor and its radiosensitization by KU60019. (**A**–**D**) Patient’s tumor at admission: axial and coronal brain MRI FLAIR images demonstrate a diffusely infiltrating lesion (arrows, **A**,**B**) involving the cortical-subcortical region of the left temporo-parietal junction without contrast enhancement (**C**,**D**, post-contrast T1-weighted images). (**E**–**H**) Patient’s tumor at progression: axial and coronal FLAIR images shows subtle diffuse infiltration of the brain parenchyma adjacent to the surgical bed and extending to the ipsilateral insular and temporal region with concomitant bi-thalamic involvement (arrows, **E**,**F**). Post-contrast T1-weighted images (**G**,**H**) do not show blood brain barrier disruption or necrotic areas. (**I**) GIC i: the GIC component of the tumor was isolated immediately after surgery. Under matrigel-coating and serum-free conditions, the cells grew and layered onto a monolayer, maintaining intact self-renewal capacity. (**J**) GIC ii: the 239/12 GIC could be effectively radiosensitized *in vitro* by exposure to 1 µM KU60019 30 min prior to irradiation with three 2.5 Gy IR fractions [blue versus black circles; reproduced from^13^, with permission]. The *in vitro* growth pattern of unirradiated cells is indicated by black squares. (**K**–**O**) Orthotopic tumor. 2 × 10^5^ 239/12 serum-free grown GIC were stereotactically injected into the left corpus striatum of NOD SCID mice. Immunostaining with an antibody directed against human nestin revealed at both d29 (**K**) and d138-150 (**M**,**P**) an infiltrating, gliomatosis-like, growth pattern of the orthotopic tumor exerting limited mass effect and reflecting the characteristics of the clinical tumor (**A**–**H**). Staining with hematoxylin/eosin of brain tissue could reveal the presence of this highly infiltrating tumor only at late times of tumor development and elevated magnification (d138; **N**,**O**). Mouse ID numbers 32.8, 32.19 and 32.14 are indicated for the sake of reference with the days (d) of tumor development. (**Q**) Beneficial effect on animal survival after RT hyperfractionation in the presence of KU60019. At d25 of tumor development, animals were i.c.-injected by CED in the surgery room with 12.5 µl of 250 µM KU60019 (blue) or vehicle (ethanol in 0.9% NaCl - black) followed by irradiation with 15 fractions of 0.5 Gy IR delivered to the head of mice 2–4 days apart from d29 until d94 (total dose delivered: 7.5 Gy). Kaplan-Meier survival curves are shown. Median survival of KU60019-treated animals was increased (186 vs 167 days; ratio: 0.8978; 95% CI of ratio: 0.5352 to 1.260; P: 0.0891) with respect to controls.

Figure 7A–H show the MRI of the 239/12 pediatric patient’s tumor. The growth pattern is that of an infiltrating, gliomatosis-like tumor that, differently from the adult COMI tumor, does not exert a significant mass effect at early stages of development. Similar to COMI, the tumor growth pattern was faithfully reproduced in the GIC-driven orthotopic tumor model. Pediatric 239/12 GIC cultured under adherent conditions in matrigel-coated flasks are shown in Fig. 7I. These cells can be radiosensitized *in vitro* by exposure to 1 µM KU60019 30 minutes prior to irradiation (Fig. 7J – blue versus black circles)^13^. The *in vitro* growth pattern of unirradiated cells is indicated by black squares. After orthotopic injection of 2 × 10^5^ 239/12 GIC in NOD SCID mice, the tumor engrafted with elevated (>95%) frequency but its progression was very slow and one month later (d29) no tumor was evident after hematoxylin/eosin staining (Fig. 7L). At this stage, immunostaining with an antibody for human nestin allowed to detect scattered, isolated tumor cells in the normal brain parenchyma (Fig. 7K). At d138-d150, a highly infiltrating tumor with little mass effect on adjacent structures could be observed after staining for nestin (Fig. 7M,P). At this late stage of development, tumor cells could be detected at elevated magnification after H/E staining as well (Fig. 7N,O). Exposure of mice bearing 239/12 orthotopic tumor to 12.5 µl of 250 µM KU60019 via CED performed at d25 of tumor development followed by 15 IR fractions of 0.5 Gy to the head delivered from d29 to d94, resulted in a trend to increased median survival as compared to mice treated by vehicle + IR (186 vs 167 days; ratio: 0.8978; 95% CI of ratio: 0.5352 to 1.260; P: 0.0891; Fig. 7Q).

## Discussion

In agreement with previous studies^13,17^, KU60019 could not cross the BBB even after chronic i.p administrations (Fig. 2). Hence, in order to exploit its remarkable and specific radiosensitizing capacity towards GIC, KU60019 was delivered i.c. to the tumor site. This was accomplished by CED^12,13^, an efficient and clinically used i.c. delivery technique^22^, allowing for steady, finely-tunable locoregional drug infusion^13,17^. CED was effective allowing significant survival improvements of mice bearing the *TP53*-deficient COMI tumor treated with one single radiosensitization cycle consisting of one KU60019 administration followed by three 2.5 Gy IR fractions (Fig. 6). Albeit those results were encouraging, being obtained in orthotopic tumors that faithfully mimic the growth pattern of the clinical tumors, the survival improvements were lower than those observed in previous studies performed on orthotopic tumors driven by the established U87 and U1242 glioma cell lines bearing specific *TP53* mutations. Several factors may have contributed to this difference. IR fractions were slightly lower (2.5 Gy) than those used by Biddlestone-Thorpe and coworkers (3 Gy) with orthotopic tumor-bearing athymic nude mice, due to the radiosensitivity of NOD-SCID mice, whose deeper immunodeficiency level is strictly required for reproducible and complete (>95%) engraftment of GIC-driven tumors^21^. Further, each cell line is different in terms of doubling time and aggressiveness and tumor volume/mass represent critical factors when different types of orthotopic tumors are compared. Lastly,.the starting of the radiosensitization was delayed (d16-d42 of tumor development) as compared to U87-U1242-driven tumors (d6-d7 of tumor development)^17^, due to the significantly slower growth rate of orthotopic GIC-driven tumors. While untreated U87-U1242 tumors usually kill xenografted mice in less than 50 days, GIC-driven tumors employ more than 90 days[^17^ Frosina *et al*., unpublished], reflecting the slower proliferation rate of GIC^7,16,19^. We believe unlikely that the above minor protocol variations may have caused the significant decrease of KU60019-mediated tumor radiosensitization observed here in comparison to the experiments reported by Biddlestone-Thorpe and coworkers^17^. The validation of ATM inhibition in the tumor by p(S1981)ATM and KI67 immunostaining under our experimental conditions (Fig. 5), supports this opinion and indicates the following three reasons as more likely explaining the discrepancy with results reported by Biddlestone-Thorpe and coworkers^17^: first, the nature and extent of the *TP53* deficiency in the orthotopic tumors used in the two studies have probably contributed to the differences. Although the COMI tumor has a low expression level of the *TP53* gene as assessed by quantitative polymerase chain reaction (qPCR)^12^, its *TP53* gene sequence is wild type thus making possible that the residual TP53 activity is sufficient to fade the *in vivo* radiosensitization response to KU60019. The highest radiosensitization efficacy of ATM inhibition by KU60019 was observed by Biddlestone-Thorpe and coworkers towards U87 tumors harboring the *TP53* D281G mutation. Since the D281G is a gain of function *TP53* mutation causing disruption of a spindle checkpoint and promoting genetic instability^23^, it might be possible that disruption of DDR checkpoints due to ATM inhibition and of spindle checkpoints linked to the D281G mutation synergized and cooperated to inhibit the tumor growth in the Biddlestone-Thorpe study. In clinical perspective, albeit we confirm that loss of TP53 function (as well as gain of PI3K function) do enhance the response to KU60019 radiosensitization^12^, we have shown by analysis of multiple patients, that even tumors with unfavorable properties (elevated *TP53* and reduced *PI3K* wild type expressions) can be radiosensitized by KU60019, although less promptly than GIC-driven tumours bearing the “responder” profile, thus making reasonable the treatment of an elevated percentage of HGG patients with ATMi^13^.

**Figure 8:**
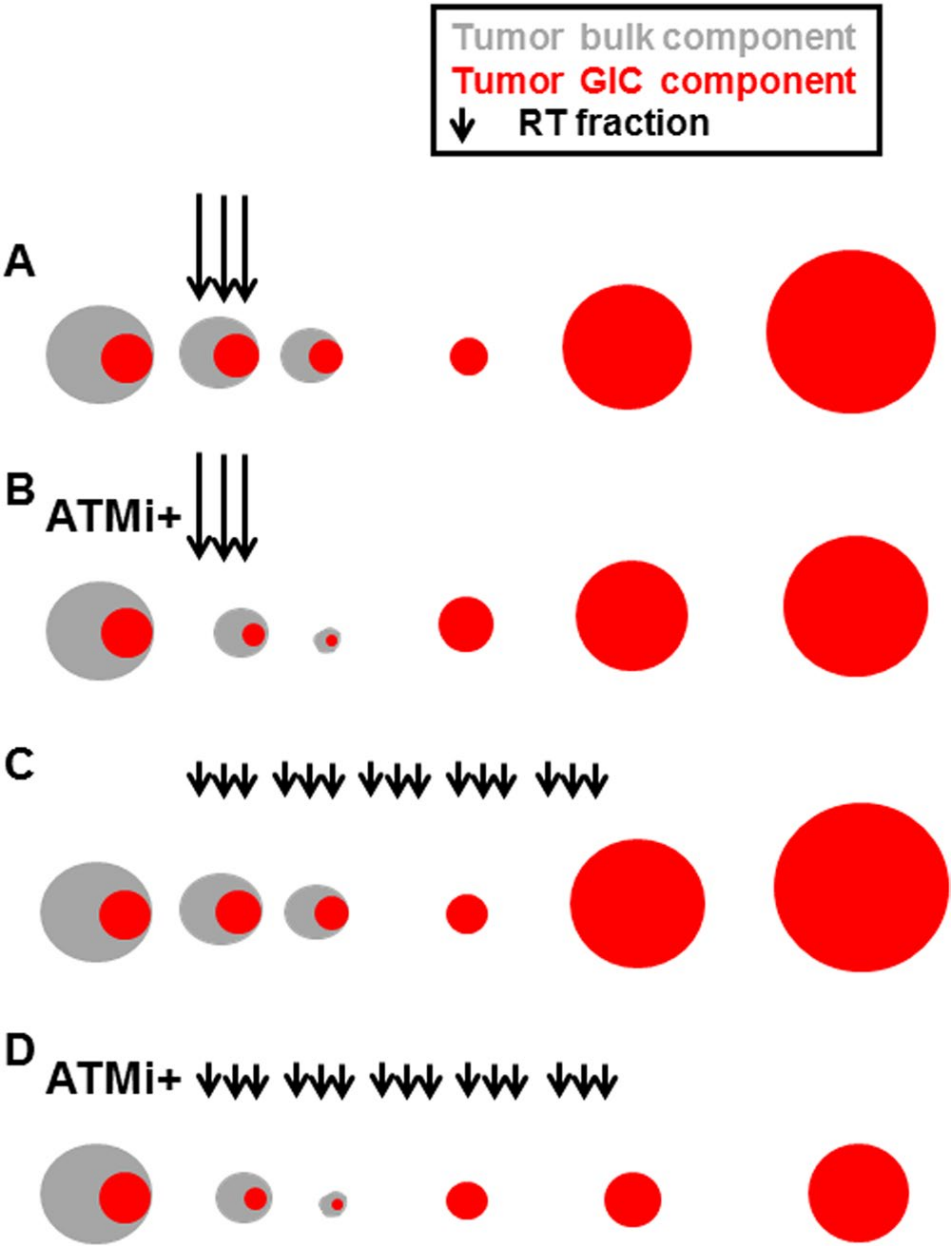
Proposed mechanism for *in vivo* radiosensitization by ATMi in GIC-driven orthotopic tumors. (**A**) RT initially exerts its limited cytocidal effect on the radioresistant, quiescent GIC component of glioma (indicated in red). Once RT is over, the GICs resume proliferation driving tumor progression. (**B**) ATMi such as KU60019 may radiosensitize the GICs and initially augment their killing by dislodging them from quiescence but, once RT is over, the persistent ATMi-mediated stimulus to proliferate causes the GIC recovery and expansion, thus mitigating the initial radiosensitization benefit. (**C**) Hyperfractionation of RT has little or no beneficial effect in limiting tumor progression as compared to standard RT fractionation in (**A**). (**D**) In the presence of ATMi, RT hyperfractionation may allow to take advantage of the long-term proliferation-boosting, radiosensitizing effect of the drug towards the GIC component of the tumor and significantly delay tumor progression.

Second, the pharmacodynamics of KU60019 may be related as well to the different responses of the two types of orthotopic tumor used in^17^ and here: the GIC proliferation-stimulating effect exerted by KU60019 that reaches a peak 96 hours (four days) after drug infusion to the orthotopic tumor^13^, may in fact sustain the growth of the GIC-driven tumors well beyond that time, as indicated by the persistent, significant increase of KI67-positive cells 42 days after the last drug infusion (d108-d66; Fig. 5C,D). The KU60019-mediated DDR inhibition may thus not be completely reversible and the prolonged stimulus to proliferate, persisting after the end of RT, may cancel most radiosensitizing benefit initially achieved on the slow-growing GIC-driven tumors (Fig. 8) (but not on the fast-growing/killing U87/U1242 tumors used in^17^). In this regard, it is not surprising that the survival benefit given by the hyperfractionated radiosensitization schedule was mitigated and did not achieve statistical significance in pediatric 239/12-bearing mice (Fig. 7Q). Because of its histopathologic features and gliomatosis-like growth mode, the 239/12 tumor did not exert considerable mass effect early after implant/diagnosis (Fig. 7A–H; K–P) and caused the death of the affected organisms more slowly than the adult COMI tumor [median survivals of vehicle + (0.5 Gy × 15) – treated mice: 167 and 89 days, respectively (88% longer in 239/12 than in COMI); post-diagnosis survival of chemo/radiotherapy-treated patients: 551 and 413 days, respectively (33% longer in 239/12 than in COMI)]. Thus, in 239/12 bearing mice, the tumor had longer time to recover aggressiveness after the last, 15^th^ radiosensitized fraction [92 days = 186 (median survival in radiosensitized 239/12 mice with 15 fractions) - 94 (day of 15^th^ RT fraction to 239/12 mice) – Fig. 7Q] as compared to COMI mice [29 days = 105 (median survival in radiosensitized COMI mice with 15 fractions) −76 (day of 15^th^ fraction to COMI mice) Fig. 6E]. In the clinical perspective, the growth properties of each tumor will have to be carefully considered in order to plan the most effective radiosensitization protocol. In this setting, the persistent pharmacodynamics effect of KU60019 on GIC-driven tumors (Fig. 5C,D) may represent a drug administration advantage: one single intraoperative KU60019 delivery will exert its radiosensitizing effects over a significant number of post-surgery adjuvant radiotherapeutic fractions^24^. If required, subsequent i.c. administrations might follow by different techniques including CED, an increasingly employed administration procedure in the clinics^22^, nanoparticle vectors^25^, permeabilization enhanced delivery and others^26^.

Third, primary GIC driven-tumors such as COMI and 239/12 maintain, similar to the clinical tumors, elevated infiltrative behavior and intratumoral heterogeneity (Figs 3H–J and 7K–P) conferring higher refractoriness to the radiosensitization procedure, as compared to U87 and U1242 tumors which are poorly infiltrating and more contained, thus providing an accessible, physical target for KU60019. Further, the DNA profile of currently used U87 is different from that of the original cells thus indicating a possible mix-up with another human GB cell line of unknown origin^27^. The use of multiple GIC-driven orthotopic tumors that faithfully mimic the patient’s tumor (Figs 3 and 7) may be essential to develop effective therapeutic protocols for HGG transferable to the clinics.

## Electronic supplementary material

Supplementary information
