# Author Correction: Faithful animal modelling of human glioma by using primary initiating cells and its implications for radiosensitization therapy

**DOI:** 10.1038/s41598-018-37928-2

**Published:** 2019-02-28

**Authors:** Guido Frosina, Jean Louis Ravetti, Renzo Corvò, Mauro Fella, Maria Luisa Garrè, Fabrizio Levrero, Diana Marcello, Daniela Marubbi, Giovanni Morana, Michele Mussap, Carlo Emanuele Neumaier, Aldo Profumo, Alessandro Raso, Francesca Rosa, Stefano Vagge, Donatella Vecchio, Antonio Verrico, Gianluigi Zona, Antonio Daga

**Affiliations:** 1 Mutagenesis & Cancer Prevention, IRCCS Ospedale Policlinico San Martino, 16132 Genova, Italy; 2 Pathological Anatomy and Histology, IRCCS Ospedale Policlinico San Martino, 16132 Genova, Italy; 3 Radiation Oncology, IRCCS Ospedale Policlinico San Martino, 16132 Genova, Italy; 4 Department of Health Sciences (DISSAL) University of Genoa, 16132 Genova, Italy; 5 Laboratory Medicine, IRCCS Ospedale Policlinico San Martino, 16132 Genova, Italy; 6 IRCCS Istituto Giannina Gaslini, 16147 Genova, Italy; 7 Medical Physics, IRCCS Ospedale Policlinico San Martino, 16132 Genova, Italy; 8 Regenerative Medicine, IRCCS Ospedale Policlinico San Martino, 16132 Genova, Italy; 9 Department of Experimental Medicine (DIMES), University of Genova, 16132 Genova, Italy; 10 Diagnostic Imaging and Interventional Oncology, IRCCS Ospedale Policlinico San Martino, 16132 Genova, Italy; 11 Biopolymers and Proteomics, IRCCS Ospedale Policlinico San Martino, 16132 Genova, Italy; 12 Neurosurgery and Neurotraumatology, IRCCS Ospedale Policlinico San Martino, 16132 Genova, Italy; 13 Department of Neurology, Ophthalmology, Genetics and Paediatrics (DINOGMI), University of Genova, 16132 Genova, Italy

Correction to: *Scientific Reports* 10.1038/s41598-018-32578-w, published online 21 September 2018

The original version of this Article contained references to the ARRIVE checklist throughout, written as ‘[ARRIVE 1]’ through to ‘[ARRIVE 20]’ inclusive. These references have now been removed from the HTML and PDF versions of this Article.

