# Supplementary material for: Faithful animal modelling of human glioma by using primary initiating cells and its implications for radiosensitization therapy
Source: Sci Rep. 2018 Sep 21;8:14191. doi: 10.1038/s41598-018-32578-w (PMC6154973; doi:10.1038/s41598-018-32578-w)
Supplement: Supplementary file 1 — Supplementary information [file 41598_2018_32578_MOESM1_ESM.pdf]

Faithful animal modelling of human glioma by using primary initiating cells and its implications for radiosensitization therapy

Guido Fròsina, Jean Louis Ravetti, Renzo Corvò, Mauro Fella, Maria Luisa Garrè, Fabrizio Levrero, Diana Marcello, Daniela Marubbi, Giovanni Morana, Michele Mussap, Carlo Emanuele Neumaier, Aldo Profumo, Alessandro Raso, Francesca Rosa, Stefano Vagge, Donatella Vecchio, Antonio Verrico, Gianluigi Zona and Antonio Daga

Supplementary Information

wildtype AA sequence	MEEPQSDPSV EPPLSQETFS DLWKLLPENN VLSPLPSQAM DDLMLSPDDI EQWFTEDPGP DEAPRMPEAA PPVAPAPAAP TPAAPAPAPS WPLSSSVPSQ KTYQGSYGFR LGFLHSGTAK SVTCTYSPAL NKMFCQLAKT CPVQLWVDST PPPGTRVRAM AIYKQSQHMT EVVRRCPHHE RCSDSDGLAP PQHLIRVEGN LRVEYLDDRN TFRHSVVVPY EPPEVGSDCT TIHYNYMCNS SCMGGMNRRP ILTIITLEDS SGNLLGRNSF EVRVCACPGR DRRTEEENLR KKGEPHHELP PGSTKRALPN NTSSSPQPKK KPLDGEYFTL QIRGRERFEM FRELNEALEL KDAQAGKEPG GSRAHSSHLK SKKGQSTSRH KKLMFKTEGP DSD*
mutated AA sequence	MEEPQSDPSV EPPLSQETFS DLWKLLPENN VLSPLPSQAM DDLMLSPDDI EQWFTEDPGP DEAPRMPEAA PPVAPAPAAP TPAAPAPAPS WPLSSSVPSQ KTYQGSYGFR LGFLHSGTAK SVTCTYSPAL NKMFCQLAKT CPVQLWVDST PPPGTRVRAM AIYKQSQHMT EVVRRCPHHE RCSDSDGLAP PQHLIRVEGN LRVEYLDDRN TFRHSVVVPY EPPEVGSDCT TIHYNYMCNS SCMGGMNRRP ILTIITLEDS SGNLLGRNSF EVRCLCLSWE RPAHRGRESP QERGASPRAA PREH*

Supplementary Figure 1. The TP53 mutation in 239/12 pediatric tumor. Reference sequence NM000546

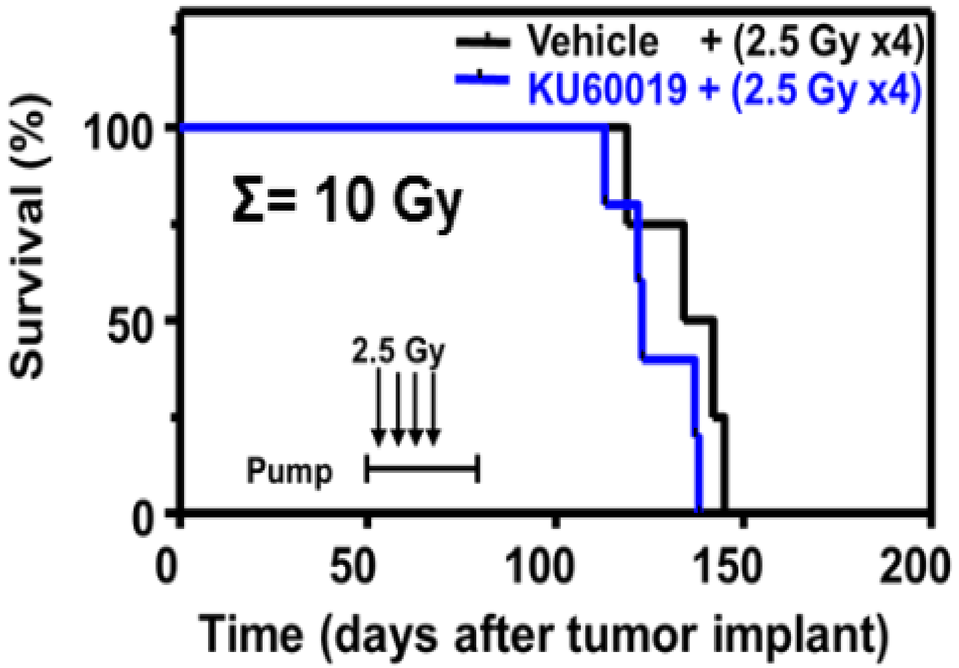

Supplementary Figure 2. Lack of survival improvement in NOD-SCID mice bearing orthotopic adult COMI GB after treatment with KU60019 + 10 Gy IR. At d50 of tumor development, animals were implanted with an Alzet osmotic pump loaded with 10,000 µM KU60019 dissolved in 10% ethanol (blue) or vehicle (black) followed at d56, 59, 63 and 66 by irradiation with 2.5 Gy (total dose delivered: 10 Gy), as previously described [12]. No significant difference in overall survival was observed between the two animal groups.
